# Minimalistic transcriptomic signatures permit accurate early prediction of COVID-19 mortality

**DOI:** 10.1172/jci.insight.195436

**Published:** 2025-11-10

**Authors:** Rithwik Narendra, Emily C. Lydon, Hoang Van Phan, Natasha Spottiswoode, Lucile P. Neyton, Joann Diray-Arce, IMPACC Network, COMET Consortium, EARLI Consortium, Patrice M. Becker, Seunghee Kim-Schulze, Annmarie Hoch, Harry Pickering, Patrick van Zalm, Charles B. Cairns, Matthew C. Altman, Alison D. Augustine, Steve Bosinger, Walter Eckalbar, Leying Guan, Naresh Doni Jayavelu, Steven H. Kleinstein, Florian Krammer, Holden T. Maecker, Al Ozonoff, Bjoern Peters, Nadine Rouphael, Ruth R. Montgomery, Elaine Reed, Joanna Schaenman, Hanno Steen, Ofer Levy, Sidney C. Haller, David Erle, Carolyn M. Hendrickson, Matthew F. Krummel, Michael A. Matthay, Prescott Woodruff, Elias K. Haddad, Carolyn S. Calfee, Charles R. Langelier

**Affiliations:** 1 UCSF, San Francisco, California, USA.; 2 UCLA, Los Angeles, California, USA.; 3 Precision Vaccines Program, Boston Children’s Hospital, Harvard Medical School, Boston, Massachusetts, USA.; 4 See Consortium Supplement A for consortium details.; 5 See Consortium Supplement B for consortium details.; 6 See Consortium Supplement C for consortium details.; 7 National Institute of Allergy and Infectious Diseases, NIH, Bethesda, Maryland, USA.; 8 Icahn School of Medicine at Mount Sinai, New York, New York, USA.; 9 Drexel University, Tower Health Hospital, Philadelphia, Pennsylvania, USA.; 10 Benaroya Research Institute, University of Washington, Seattle, Washington, USA.; 11 Emory School of Medicine, Atlanta, Georgia, USA.; 12 Yale School of Public Health, New Haven, Connecticut, USA.; 13 Yale School of Medicine, New Haven, Connecticut, USA.; 14 Stanford University School of Medicine, Palo Alto, California, USA.; 15 La Jolla Institute for Immunology, La Jolla, California, USA.; 16 Chan Zuckerberg Biohub San Francisco, San Francisco, California, USA.

**Keywords:** Infectious disease, Pulmonology, Biomarkers, COVID-19, Machine learning

## Abstract

**BACKGROUND:**

Accurate prognostic assays for COVID-19 represent an unmet clinical need. We sought to identify and validate early parsimonious transcriptomic signatures that accurately predict fatal outcomes.

**METHODS:**

We studied 894 patients enrolled in the prospective, multicenter Immunophenotyping Assessment in a COVID-19 Cohort (IMPACC) with peripheral blood mononuclear cells (PBMC) and nasal swabs collected within 48 hours of admission. Host gene expression was measured with RNA-Seq. We trained parsimonious prognostic classifiers incorporating host gene expression, age, and SARS-CoV-2 viral load to predict 28-day mortality in 70% of the cohort. Classifier performance was determined in the remaining 30% and externally validated in a contemporary COVID-19 cohort (*n* = 137) with vaccinated patients.

**RESULTS:**

Fatal COVID-19 was characterized by 4,189 differentially expressed genes in the peripheral blood. A COVID-specific 3-gene peripheral blood classifier (*CD83*, *ATP1B2*, *DAAM2*) combined with age and SARS-CoV-2 viral load achieved an area under the receiver operating characteristic curve (AUC) of 0.88 (95% CI, 0.82–0.94). A 3-gene nasal classifier (*SLC5A5*, *CD200R1*, *FCER1A*), in comparison, yielded an AUC of 0.74 (95% CI, 0.64–0.83). Notably, *OLAH*, the most strongly upregulated gene in both PBMC and nasal swab and recently implicated in severe viral infection pathogenesis, yielded AUCs of 0.86 (0.79–0.93) and 0.78 (95% CI, 0.69–0.86), respectively. Both peripheral blood classifiers demonstrated comparable performance in an independent contemporary cohort of vaccinated patients (AUCs 0.74–0.80).

**CONCLUSION:**

Our parsimonious blood- and nasal-based classifiers accurately predicted COVID-19 mortality and merit further study as accessible prognostic tools to guide triage, resource allocation, and early therapeutic interventions.

**FUNDING:**

NIH: 5R01AI135803-03, R35HL140026, 5U19AI118608-04, 5U19AI128910-04, 4U19AI090023-11, 4U19AI118610-06, R01AI145835-01A1S1, 5U19AI062629-17, 5U19AI057229-17, 5U19AI125357-05, 5U19AI128913-03, 3U19AI077439-13, 5U54AI142766-03, 5R01AI104870-07, 3U19AI089992-09, 3U19AI128913-03, 5T32DA018926-18, and K0826161611. National Institute of Allergy and Infectious Diseases, NIH: 3U19AI1289130, U19AI128913-04S1, and R01AI122220. National Center for Advancing Translational Sciences, NIH: UM1TR004528. The National Science Foundation: DMS2310836. The Chan Zuckerberg Biohub San Francisco.

## Abstract 2

A three gene peripheral blood signature, as well as *OLAH* alone, accurately predict COVID-19 mortality early in hospitalization, including in vaccinated patients.

## Introduction

The clinical course of SARS-CoV-2 infection is highly heterogeneous, ranging from minimal symptoms to fatal disease (1, 2). Despite thousands of studies since the emergence of the virus in 2019 (3) and a growing understanding of the biological features underpinning severe COVID-19 (4–6), clinicians still lack reliable prognostic assays to identify which patients will progress to critical illness or fatal disease. Accurate and timely severity prediction tools could improve clinical triage, optimize resource allocation, and have utility for predictive enrichment in clinical trials of novel therapeutics (7–10).

Host factors including age (11) and individual inflammatory responses are key determinants of disease severity and progression (11–14). Broadly available clinical laboratory tests, such as ferritin, D-dimer, lactate dehydrogenase, troponin, IL-6 and IL-8 have been used to risk stratify patients with COVID-19, but each biomarker individually has limited performance (15, 16). Bioinformatic approaches attempted to integrate these laboratory values with clinical parameters, resulting in modest improvements in predictive ability (17–20). However, these studies have generally been single-institution studies, leveraging a small list of biomarkers, with concerns about model overfitting and lack of generalizability (21). No models have yet been implemented into an actionable, widely used prognostic tool in clinical practice.

High-dimensional host transcriptional profiling offers a broad, systems-level view of the immune response to infection that may not be fully captured by predefined protein biomarker panels (22). Transcriptomic classifiers have increasingly shown promise in accurately diagnosing infection and predicting disease severity across a wide range of pathogens (23–27). A handful of early foundational studies, based on relatively small cohorts of ≤ 100 patients, have explored using host transcriptomic classifiers to predict COVID-19 severity (28–31).

For instance, from a preexisting panel of 29 genes, a 6-gene prognostic classifier trained on blood transcriptomic data from non-SARS-CoV-2 viral infections was developed, which—when tested in patients with COVID-19—achieved AUCs ranging from 0.65 to 0.89 (28, 29). Similarly, another group repurposed a 10-gene sepsis mortality prediction score and found that it achieved an AUC of 0.86 in patients with COVID-19 (30), and a third developed a 48-gene prognostic classifier that had an overall accuracy of 81% (31).

While these early, important studies suggest that a transcriptomic COVID-19 severity classifier has potential, there remains an unmet need for a rigorously validated, clinically translatable mortality prediction tool, deployable at the time of hospitalization, with generalizability to diverse populations that include COVID-19–vaccinated individuals. Notably, all previously published classifiers rely on sizable multigene combinations, while highly parsimonious (≤3 gene) classifiers have not yet been identified. Minimal gene expression models could enhance feasibility for clinical translation, reduce assay costs, and improve accessibility in resource-limited settings. Furthermore, compact gene signatures could be more readily incorporated into existing SARS-CoV-2 diagnostic platforms, facilitating rapid risk stratification at the time of diagnosis.

Here, we addressed this need by studying over 1,000 patients with COVID-19 enrolled in 2 cohorts across 20 hospitals in the United States. We identified single- and 3-gene signatures from peripheral blood and nasal swabs collected within 48 hours of hospital admission that accurately predicted future COVID-19 mortality, including in vaccinated patients. We further demonstrated that incorporating patient age and SARS-CoV-2 viral load enhances prognostic ability of transcriptomic classifiers, offering what we believe to be a novel, translatable approach for early risk stratification in hospitalized patients with COVID-19.

## Results

### Clinical and demographic features associated with fatal COVID-19.

**Figure 1:**
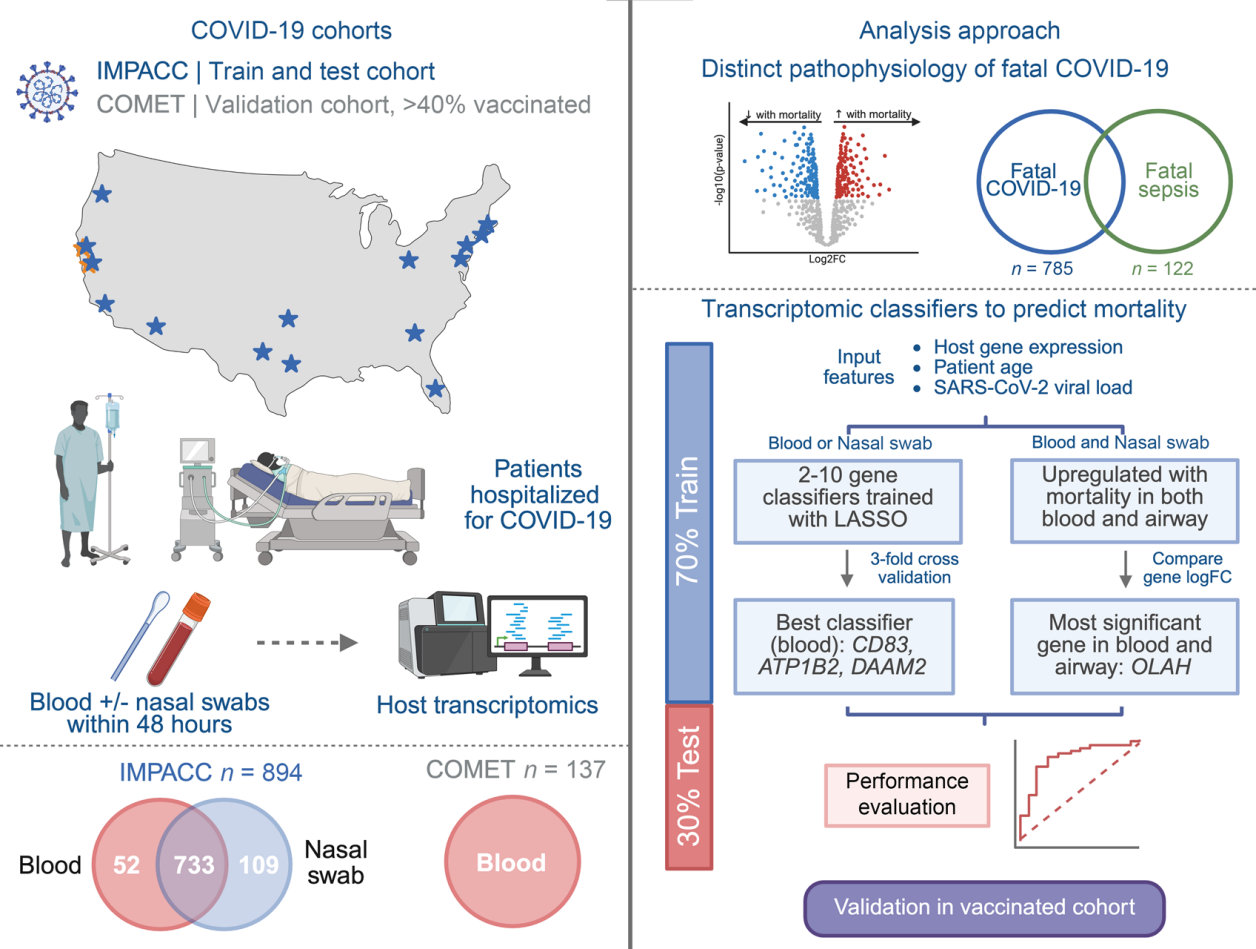
Overview schematic of study. This study evaluated 894 patients hospitalized with COVID-19 from the multicenter IMPACC cohort. Peripheral blood and nasal swab samples collected within 48 hours of hospitalization were utilized to evaluate host transcriptional signatures of mortality, which were then compared with sepsis mortality signatures. Parsimonious mortality prediction classifiers of varying lengths were then developed on 70% of the data (training cohort), and performance characteristics were assessed in the remaining held-out 30% of the data (test cohort). Classifiers were then validated in an external cohort with vaccinated patients, and performance was compared with other larger classifiers published in the literature. Figure created in BioRender. IMPACC, ImmunoPhenotyping Assessment in a COVID-19 Cohort; COMET, COVID-19 Multi-Immunophenotyping Projects for Effective Therapies; QC, quality control; LASSO, least absolute shrinkage and selection operator; logFC = log gene expression fold change.

We analyzed 894 patients enrolled in the Immunophenotyping Assessment in a COVID-19 cohort (IMPACC) who had peripheral blood and/or nasal swab samples collected at early time points in their hospitalization, as well as SARS-CoV-2 viral load measured in the upper airway (Figure 1). We began by first evaluating the demographic and clinical features of fatal SARS-CoV-2 infection (Table 1). The overall mortality rate was 9.5%. Consistent with many prior studies, older age strongly correlated with mortality in patients with COVID-19 from IMPACC (median 70.0 in mortality versus 58.0 in survival, *P* < 0.001), as did higher viral load, reflected by a lower quantitative PCR (qPCR) cycle threshold (CT) value (median CT 25.5 in mortality versus 27.6 in survival, *P* = 0.002). Patients who did not survive had a higher oxygen requirement on presentation (*P* < 0.001), though both survival and mortality groups shared a wide range. Higher Sequential Organ Failure Assessment (SOFA) (32) scores (*P* < 0.001), higher C-reactive protein (CRP) (*P* = 0.003), and lower absolute lymphocyte count (ALC) (*P* < 0.001) on admission were each associated with mortality. Most comorbidities that were evaluated were associated with mortality, including hypertension, diabetes, chronic lung disease, cardiovascular disease, chronic kidney disease, and malignancy. Therapeutically, steroid use was higher in patients who did not survive (81% versus 66%, *P* = 0.005), though remdesivir use was similar (61% versus 63%, *P* = 0.735). As patients were enrolled between May 2020 and March 2021, this cohort was unvaccinated.

### Early host transcriptional signatures of fatal COVID-19 exist in the blood and upper respiratory tract.

**Figure 2:**
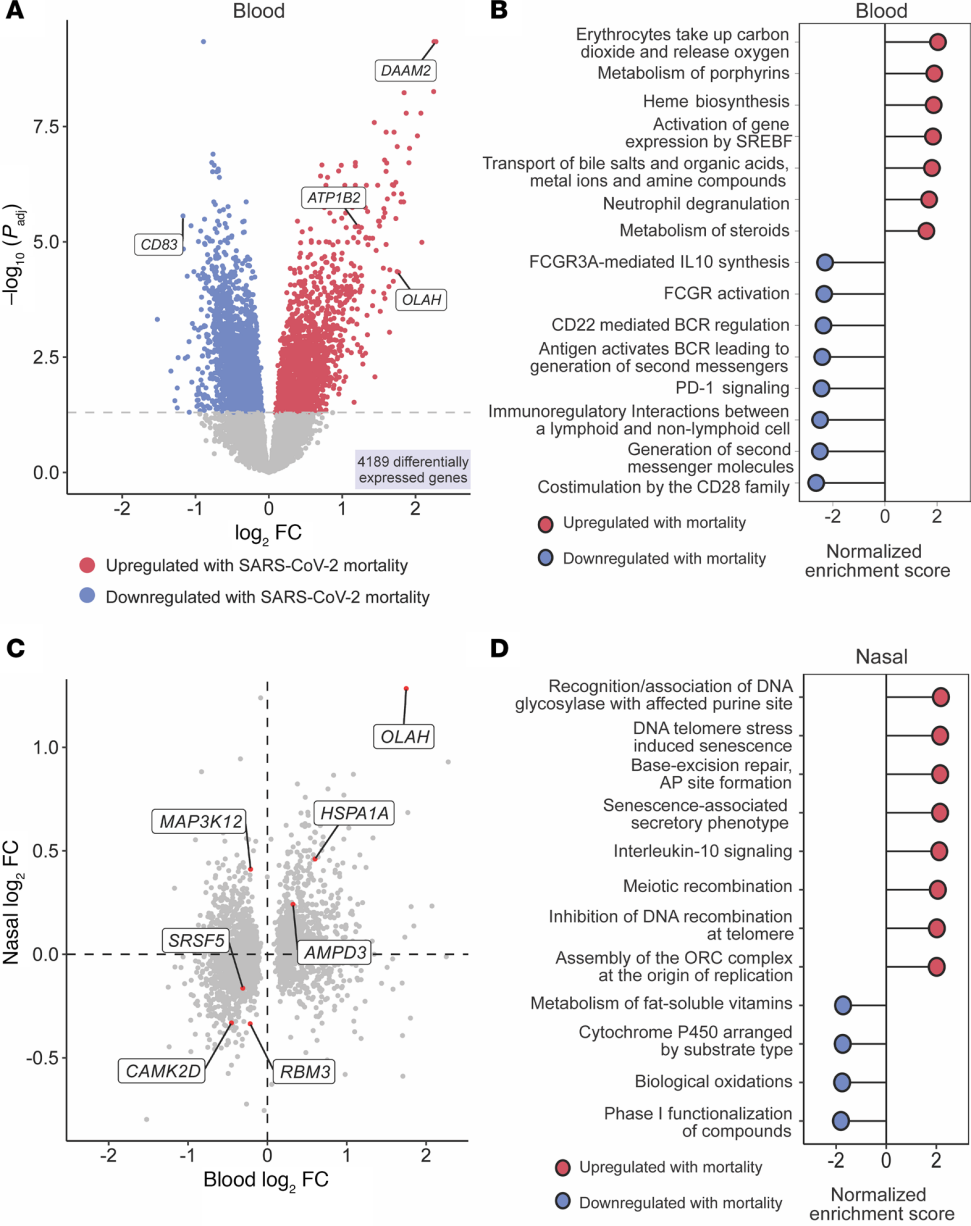
Early host gene expression signatures of mortality in the peripheral blood and upper airway. (**A**) Volcano plot displaying the 4,189 genes that were differentially expressed (DE) between survival and mortality in the peripheral blood of patients with COVID-19, using a Benjamini-Hochberg *P*_adj_ < 0.05. Genes part of prognostic classifiers are labeled. (**B**) Gene set enrichment analysis (GSEA) demonstrating statistically significant pathways associated with mortality based on Benjamini-Hochberg *P*_adj_ in the peripheral blood (red, upregulated with mortality; blue, downregulated with mortality). (**C**) Log-log plot demonstrating the 7 genes that were DE in both peripheral blood and nasal swab samples. log_2_FC = base 2 logarithm of fold change. (**D**) GSEA demonstrating the differentially expressed mortality pathways in nasal samples (red, upregulated pathways; blue, downregulated pathways).

We next evaluated the relationship between peripheral blood mononuclear cell (PBMC) gene expression profiles within 48 hours of hospital admission and COVID-19 mortality within 28 days (*n* = 785). We identified 4,189 differentially expressed (DE) genes (adjusted *P* value [*P*_adj_] < 0.05), adjusting for sex and race (Figure 2A and Supplemental Data 1A; supplemental material available online with this article; https://doi.org/10.1172/jci.insight.195436DS1). To explore their functions, we performed gene-set enrichment analysis (GSEA). Patients who died exhibited upregulation in genes related to erythrocyte gas exchange (e.g., *CA1* and *CA4*), heme biosynthesis (e.g., *HBA1*, *HBA2*, and *FECH*), neutrophil degranulation (e.g., *MPO* and *TNF*), among other pathways (Figure 2B and Supplemental Data 1B). This was juxtaposed against downregulated expression of genes important for adaptive immunity, including B and T lymphocyte signaling (e.g*., CD22*, *CD79*, *CD96*, and *CD4*).

We performed a similar analysis of transcriptomic data derived from nasal swabs collected within 48 hours of hospital admission (*n* = 842). A host signature of mortality was also present in the upper respiratory tract, although differential gene expression was more subtle, with only 53 genes significantly associated with mortality (Supplemental Figure 1A and Supplemental Data 2A). Of these, 7 were consistently DE across both the peripheral blood and the upper airway, with *OLAH*, which encodes oleoyl-ACP hydrolase, most strongly upregulated with mortality in both nasal swab and PBMC samples (Figure 2C). In the upper airway, mortality was associated with nucleic acid repair, cellular senescence, and IL-10 signaling pathways (Figure 2D and Supplemental Data 2B).

### The transcriptional signature of fatal COVID-19 has unique features compared with fatal sepsis.

**Figure 3:**
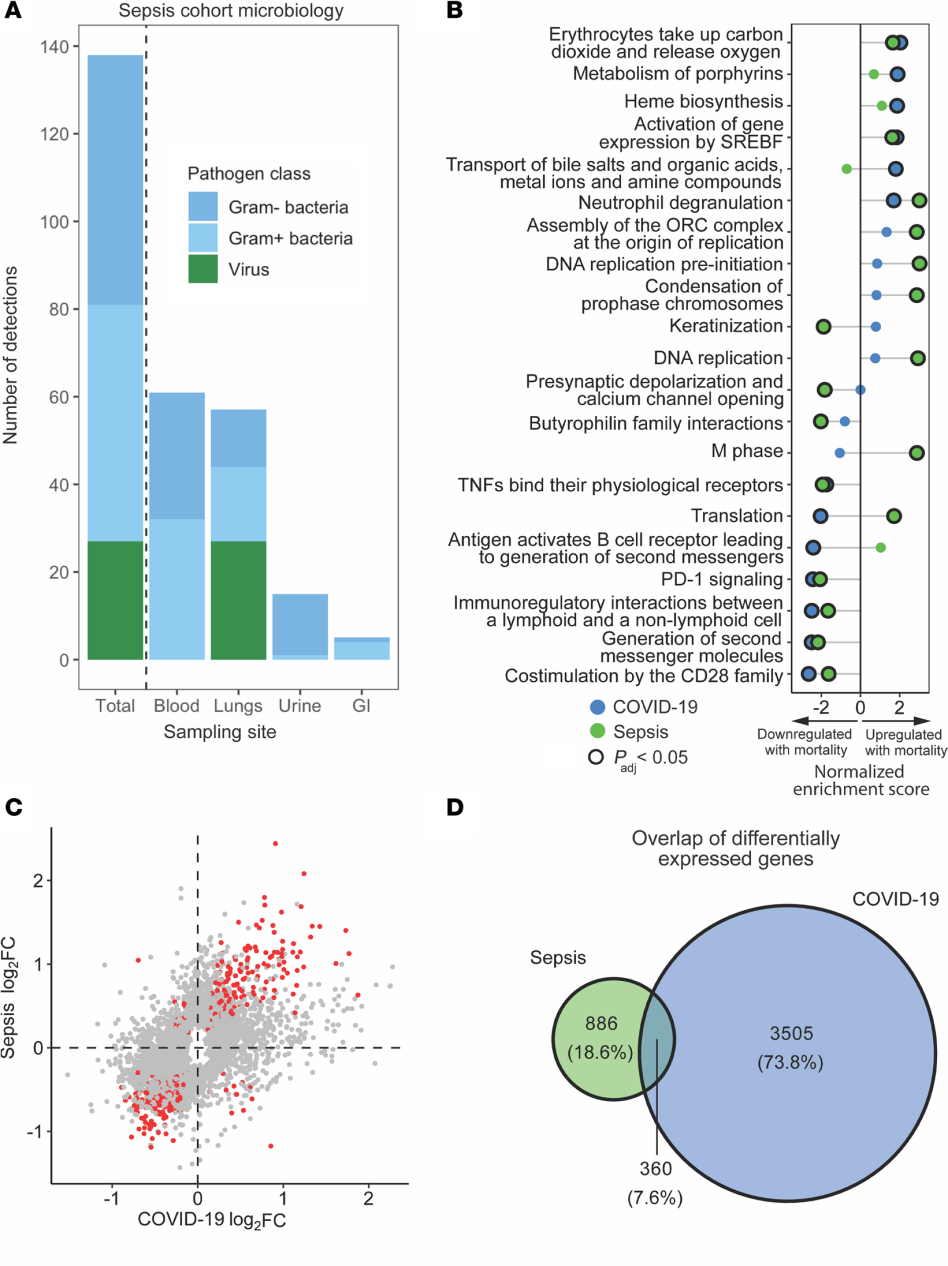
Comparison of mortality signatures between COVID and non–COVID-19 sepsis. (**A**) Microbiology of the non–COVID-19 sepsis cohort (*n* = 122), stratified by sampling site and pathogen category. The total bar on the left shows the summation of the bars to the right, and the number of detections exceeds the number of patients as some patients had multiple pathogen classes detected. (**B**) Gene set enrichment analysis (GSEA) of COVID-19 mortality (blue circles) overlaid that of sepsis mortality (green circles). Circles outlined in black are statistically significant based on Benjamini-Hochberg *P*_adj_. (**C**) Log-log plot of differentially expressed genes in COVID-19 and sepsis mortality, with genes statistically significant in both (based on *P*_adj_) highlighted in red. (**D**) Venn diagram highlighting the limited overlap (7.6%) between DE genes in COVID-19 and sepsis.

Understanding whether the host response leading to death in COVID-19 is distinct from or shared with other forms of sepsis could provide insights into disease-specific mechanisms or risk stratification strategies. We therefore sought to determine whether host transcriptional signatures of mortality early in hospital admission were similar or different between patients hospitalized for COVID-19 versus sepsis due to other causes. To address this question, we analyzed peripheral whole blood RNA-Seq data from 122 patients hospitalized for microbiologically confirmed sepsis prior to the COVID-19 pandemic, a cohort that had a 34.4% mortality rate and a predominance of bacterial infections (Figure 3A and Supplemental Table 1) (33–35). We identified a distinct host signature of sepsis mortality characterized by 1,246 DE genes (Supplemental Data 3A).

At the biological pathway level, GSEA suggested that fatal COVID-19 and non–COVID-19 sepsis were both characterized by increased expression of neutrophil degranulation genes and downregulation of T cell signaling genes (Figure 3B and Supplemental Data 3B). Fatal COVID-19, however, seemed uniquely characterized by impaired expression of genes related to B cell signaling and translation and by increased expression of genes functioning in heme biosynthesis. Differences between fatal COVID-19 and non–COVID-19 sepsis were even more apparent at the individual gene level (Figure 3C), with only 360 (7.6%) of mortality-associated genes shared between groups (Figure 3D). Taken together, these results demonstrate that fatal SARS-CoV-2 infection had unique transcriptional changes compared with sepsis caused by other pathogens, suggesting that accurate prognostic assessment for COVID-19 warrants a classifier specifically trained on these distinctive COVID-19 mortality signatures rather than relying on classifiers developed for other critical illnesses.

### Parsimonious host-viral classifiers accurately predict COVID-19 mortality.

Given the striking transcriptomic signature of COVID-19 mortality, we next sought to build prognostic classifiers based on gene expression measured within the first 48 hours of hospitalization. To maximize potential for future clinical translation, we sought to identify parsimonious feature sets of ≤ 10 genes. For derivation of the classifiers, we divided the cohort into training (70% of patients) and test sets (30%) (Figure 1). Given that age and SARS-CoV-2 viral load (qPCR CT value) are well established risk factors for fatal COVID-19 and readily obtainable from all hospitalized patients, we included both as additional parameters in the models.

**Figure 4:**
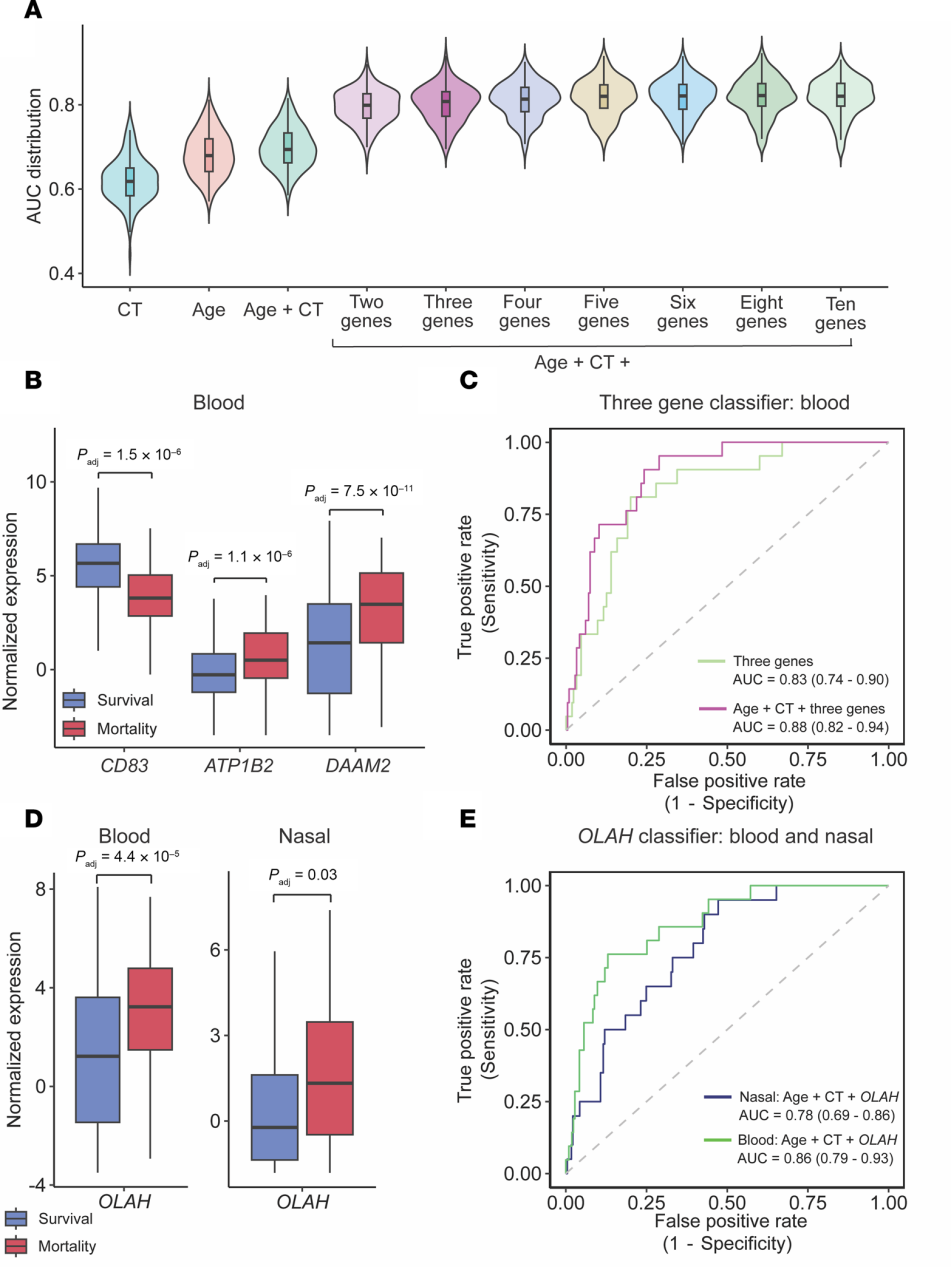
Parsimonious host-viral classifiers predict COVID-19 mortality. (**A**) Violin plots showing the area under the curve (AUC) distribution for each of the peripheral blood candidate classifiers, evaluated in the training cohort. Violin plots showing the performance of SARS-CoV-2 CT, age, and age + CT are displayed. (**B**) Box plots comparing the log_2_ counts per million normalized gene expression of the 3 genes in the optimally performing classifier between survival (blue) and mortality (red) in the full cohort. (**C**) Receiver operating characteristic (ROC) curves for the 3-gene classifier, alone and with the addition of age + CT, as evaluated in the test set (AUC ± 95% CI). (**D**) Box plots comparing the log_2_ counts per million normalized gene expression for *OLAH* in blood and nasal swab between survival (blue) and mortality (red) for the full cohort. (**E**) ROC curves for OLAH classifiers with the addition of age and CT value in the test set (AUC ± 95% CI). All *P*-values (*P*_adj_) shown were adjusted for multiple comparisons using the Benjamini-Hochberg method.

We first used least absolute shrinkage and selection operator (LASSO) regression to build 2–10 gene peripheral blood classifiers within the training set (Supplemental Table 2). These gene sets were combined in a logistic regression model with age and SARS-CoV-2 CT value, and performance distribution was assessed using a 3-fold repeated random partitioning approach (Figure 4A). We found that classifier performance plateaued at a classifier size of 3 genes, with the combination of *CD83, ATP1B2*, and *DAAM2* performing as well as the larger gene sets (Figure 4B). *CD83* plays a role in the activation of B cells and DCs (36, 37); *ATP1B2* is a component of sodium-potassium pumps that are important for maintaining endothelial integrity (38); and *DAAM2* regulates the Wnt signaling pathway, thereby influencing cell fate (39). When tested in the held-out 30% validation set, this 3-gene classifier achieved an AUC of 0.88 (95% CI, 0.82–0.94) (Figure 4C).

Using the same methodology, we derived classifiers using nasal swab transcriptomic data in the training set (Supplemental Figure 1B and Supplemental Table 3). However, the best performing 3-gene set (*SLC5A5*, *CD200R1, FCER1A*; Supplemental Figure 1C) only achieved an AUC of 0.74 (95% CI, 0.64–0.83) when evaluated on the held-out test set (Supplemental Figure 1D).

Given that *OLAH* expression was conspicuously amplified in fatal COVID-19 both in the upper respiratory tract and blood (Figure 3C and Figure 4D), and because *OLAH* was recently implicated in the pathogenesis of severe viral pneumonia (40), we also evaluated its performance in a single-gene classifier. When assayed in the blood, in combination with age and SARS-CoV-2 CT value, *OLAH* remarkably achieved an AUC of 0.86 (0.79–0.93) (Figure 4E). When assessed in the upper airway, an *OLAH* prognostic classifier achieved an AUC of 0.78 (0.69–0.86) (Figure 4E). When we tested the combination of the 3-gene classifiers and *OLAH*, both in PBMC and nasal swab, the combined performance did not exceed each individually, likely because the features are redundant with respect to classifier performance (Supplemental Figure 2). Taken together, these findings demonstrated that 1–3 gene parsimonious classifiers from either blood or nasal swab samples can accurately predict future COVID-19 mortality.

### Validation in an independent cohort with vaccinated patients with COVID-19.

**Figure 5:**
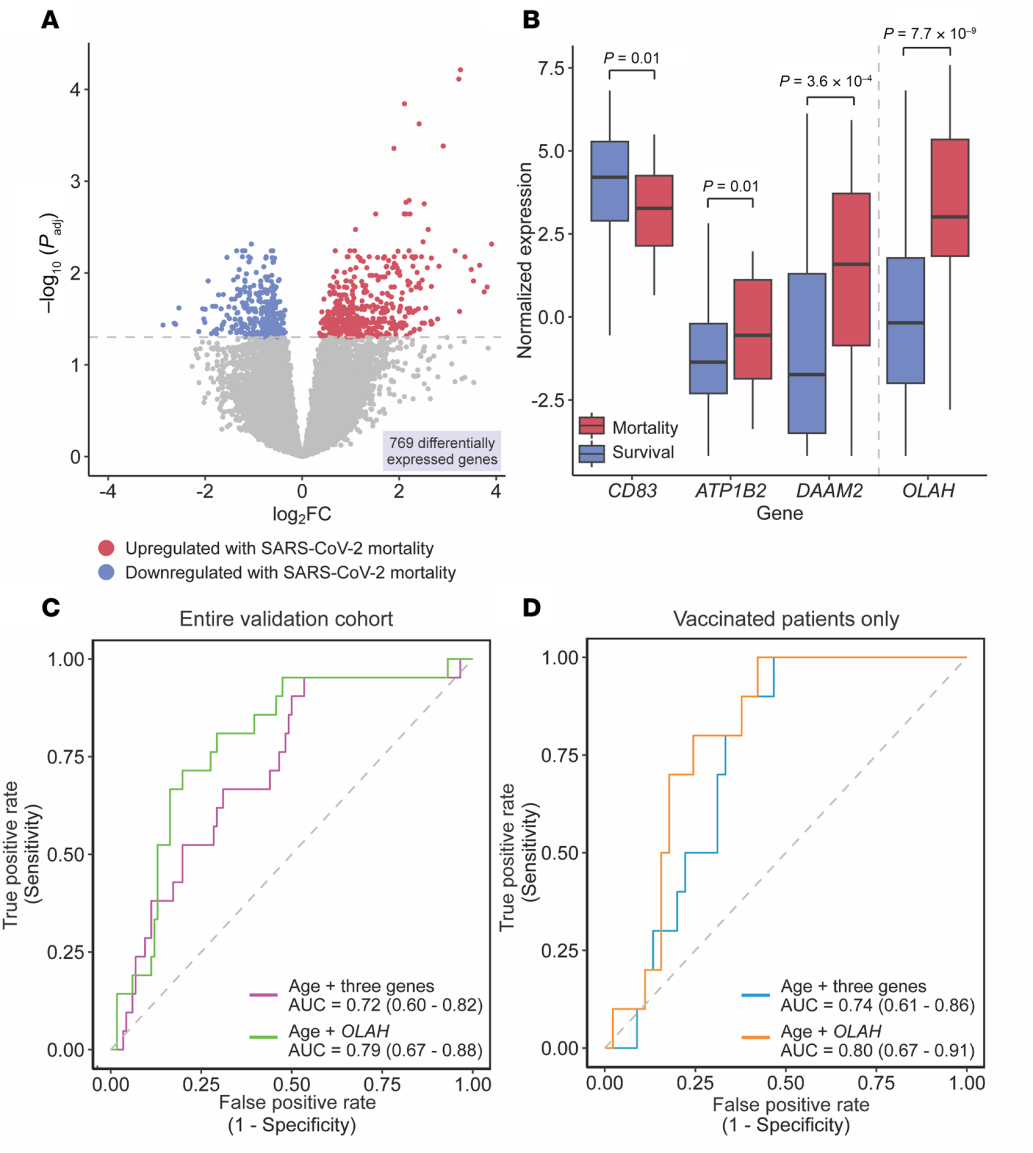
Validation of COVID-19 mortality signature and host-viral classifiers in an independent cohort including vaccinated patients. (**A**) Volcano plot demonstrating the 769 differentially expressed genes between mortality and survival in the validation cohort (red, upregulated with mortality; blue, downregulated with mortality), using a Benjamini-Hochberg *P*_adj_ < 0.05. (**B**) Box plots comparing the log_2_ counts per million normalized gene expression of the 3-gene classifier genes (*CD83*, *ATP1B2*, *DAAM2*) and single-gene OLAH classifier between survival (blue) and mortality (red) in the external validation data set. (**C**) Performance of 3-gene classifier (purple) and single-gene classifier (green) with the added feature of age in the validation cohort. Area under the curve (AUC) listed as value ± 95% CI. (**D**) Performance of 3-gene classifier (orange) and single-gene classifier (teal) with the added feature of age in the validation cohort in vaccinated patients only.

We next explored the extent to which our findings were generalizable. To that end, we leveraged the COVID-19 Multi-Immunophenotyping Projects for Effective Therapies (COMET) cohort, which enrolled patients with COVID-19 (PBMC, *n* = 137) at 2 hospitals through 2023 and notably included 55 (40.1%) vaccinated patients (Supplemental Table 4) (41). Differential expression analysis yielded 769 DE genes, confirming a robust peripheral blood signature of mortality in this validation cohort (Figure 5A and Supplemental Data 4A). GSEA demonstrated that neutrophil degranulation and erythrocyte transport of oxygen and carbon dioxide remained 2 of the most significantly upregulated pathways with mortality, but it showed a notable absence of significantly downregulated adaptive immunity pathways (Supplemental Figure 3 and Supplemental Data 4B).

Despite some minor differences at the biological pathway level, the expression of *OLAH* as well as *CD83*, *ATP1B2*, and *DAAM2* differed significantly (*P* < 0.05) based on mortality in the validation cohort (Figure 5B). Because SARS-CoV-2 CT value was not available on COMET patients, we tested the genes in combination with just age. Using 5-fold cross validation followed by out-of-fold AUC calculation, *OLAH* achieved an AUC of 0.79 (0.67–0.88), and the 3-gene classifier an AUC of 0.72 (0.60–0.82) (Figure 5C). When repeating this for vaccinated patients, the 3-gene and *OLAH* classifiers performed equally well, if not better, at predicting mortality in the vaccinated subset (Figure 5D). Collectively, these findings demonstrated that these transcriptomic classifiers remained capable of predicting mortality in an independent cohort inclusive of vaccinated individuals.

### Parsimonious single- and 3-gene prognostic classifiers outperform clinical variables and rival a larger multigene classifier.

Next, we sought to compare our classifier performance against both conventional clinical predictors and a previously published, larger host-based classifier. First, we tested clinical prognostic variables—including baseline respiratory ordinal score, SOFA score, CRP, and ALC—for mortality prediction, which achieved AUCs ranging from 0.59–0.71 (Supplemental Figure 4). When compared head-to-head with SOFA, a widely established clinical prognostic score, each of our PBMC classifiers exhibited statistically superior performance (Supplemental Table 5). Furthermore, when we added SOFA to our PBMC classifiers, performance did not significantly exceed the classifiers alone, suggesting that our classifiers already capture the majority of predictive information imparted by SOFA. Finally, we compared the results of our single-gene *OLAH* classifier and our 3-gene classifier to a previously published 6-gene classifier (*HK3*, *LY86*, *TGFB1*, *DEFA4*, *BATF*, and *HLA-DPB1*) that was developed in non–COVID-19 viral infections and previously tested in patients with COVID-19 (28). These 6 genes trained and tested in IMPACC yielded an AUC of 0.75, which improved to 0.88 after including age and CT value (Supplemental Figure 5). The performance of the published 6-gene was comparable with our integrated 3-gene and single-gene *OLAH* classifier, suggesting that 1- and 3-gene classifiers can perform comparably with larger size classifiers and that adding age and viral load can boost the performance of existing classifiers for COVID-19 risk stratification.

## Discussion

In a large, prospective, multicenter cohort, we find that either a single gene, *OLAH,* or a combination of 3 genes, *CD83*, *ATP1B2*, and *DAAM2*, accurately predicted 28-day mortality in hospitalized patients with COVID-19, including those who have been vaccinated. We built on extensive foundational studies establishing clinical and biological risk factors for severe COVID-19 (12, 42–44) by characterizing early host transcriptional determinants of survival versus death in comparison with hospitalized patients with non–COVID-19 sepsis. We then leveraged these findings to build parsimonious host-based classifiers from both blood and nasal swab samples that could be adapted to existing nucleic acid amplification platforms for clinical deployment.

The 3-gene classifier achieved an AUC of 0.88 (0.82–0.94) and remarkably, the single-gene *OLAH* classifier achieved an AUC of 0.86 (0.79–0.93) in the blood and 0.78 (0.69–0.86) in nasal swab samples. Similar performance was observed in an independent validation cohort, which included vaccinated patients, and mortality prediction was similar when stratifying by vaccinated status. Several prior studies, representing important early contributions, developed severity or mortality prediction classifiers for COVID-19 (28–31, 45). Each, however, was limited by small sample sizes, single-institution cohorts, development in non–COVID-19 populations, and testing in unvaccinated patients. These classifiers incorporated anywhere from 6 to 48 genes, whereas we identified single and 3-gene classifiers that achieved equivalent performance in head-to-head comparisons. Additionally, when compared with SOFA, a widely used mortality prediction tool, our classifiers showed superior performance while also capturing the majority of predictive information imparted by SOFA. Therefore, rather than needing to integrate multiple clinical variables, clinicians could rely on a single high-performing tool, alongside their clinical judgment, in triage and decision-making.

Beyond their prognostic potential, each of the classifier genes we report have been previously linked to COVID-19 in the literature (46–48), suggesting that they may reflect biologically relevant aspects of disease pathogenesis. *CD83*, a well-established regulator of immune responses that promotes T and B cell maturation, was downregulated in fatal COVID-19 (49), consistent with impaired adaptive immunity. *ATP1B2* encodes a regulatory subunit of the sodium/potassium-transporting ATPase pump, and its dysregulation may disrupt vascular endothelial (38, 50) and alveolar epithelial integrity (51), promoting capillary leak and acute respiratory distress syndrome. *DAAM2* negatively regulates Wnt signaling (52), a pathway implicated in the activation of inflammatory macrophages (53), angiogenesis (54), endothelial integrity (55), and fibrosis (56). Elevated *DAAM2* has been linked with vascular disorders of pregnancy (39, 57), suggesting it may also contribute to the widespread vascular dysfunction observed in fatal COVID-19 (58). Though interesting, we note that the causal role of these genes in COVID-19 pathogenesis remains speculative and requires further investigation.

*OLAH* (oleoyl-acyl-carrier-protein hydrolase), an enzyme involved in fatty acid biosynthesis, was recently implicated in the pathogenesis of life-threatening respiratory viral infections (40). *OLAH*-KO mice demonstrated protection against severe influenza infection, reduced inflammatory damage, and improved control of viral replication, outcomes attributed to modulating lipid mediators of inflammation (40). Importantly, *OLAH* expression was found to be increased across patients with severe influenza virus, RSV, and SARS-CoV-2 infection (40), suggesting that an *OLAH* prognostic classifier may be generalizable across a diversity of respiratory viral infections. Here, we independently validated the association of both airway and peripheral blood *OLAH* expression with COVID-19 severity, and we provide what we believe to be the first assessment of its performance as a prognostic biomarker in 2 large cohorts of hospitalized patients.

A growing body of literature demonstrates that severe COVID-19 is driven by a profoundly dysregulated host immune response to the virus, characterized by excessive innate inflammation and impaired adaptive immunity (5, 59). Hyperactive neutrophils and macrophages contribute to cytokine release, complement activation, endothelial damage, and vascular thrombosis, while impaired lymphocyte responses delay viral clearance and increase vulnerability to secondary infections (60–67). In our study, the systemic host signature of fatal COVID-19 mirrored many of the same pathways described previously, including increased neutrophil degranulation, decreased production of the antiinflammatory cytokine IL-10, and downregulated T cell and B cell signaling — highlighting that this aberrant immune signaling begins early in the course of illness. In addition, aligning with prior studies (68), we noted increased systemic expression of erythrocyte gas exchange and heme biosynthesis genes in fatal cases, which could reflect a compensatory mechanism for severe hypoxemia (69, 70).

The host signature of mortality in the upper respiratory tract, in contrast, was dominated by upregulation of DNA repair and senescence pathways, potentially reflecting heightened cellular stress and direct damage at the site of viral entry (71, 72). Intriguingly, IL-10 signaling was upregulated in the upper respiratory tract but downregulated systemically, suggesting a localized attempt to control inflammation that fails to extend to the systemic immune response (73, 74).

Many of the pathways enriched in fatal COVID-19 have also been described in fatal sepsis (75, 76). Prior studies have suggested that critically ill patients may follow a common mortality trajectory, with 1 study reporting that COVID-19 and patients without COVID-19 admitted to the ICU for greater than 7 days were almost transcriptionally indistinguishable (77). While we did find overlapping mortality-associated signaling pathways when comparing our GSEA results against those from a cohort of primarily bacterial sepsis patients (e.g., upregulated neutrophil degranulation, downregulated T cell signaling), more than 90% of mortality-associated genes differed between COVID-19 and sepsis. Fatal COVID-19 was uniquely characterized by decreased expression of B cell signaling genes and enrichment of heme biosynthesis pathways. These findings suggest that, while mortality pathways may eventually converge, pathogen-specific mortality signatures are prominent early in the course of severe disease — an important consideration for developing accurate early mortality prediction tools. Though the non–COVID-19 sepsis and COVID-19 sepsis cohorts differed in sample type (whole blood and PBMCs, respectively), comparative transcriptomic studies have demonstrated that gene expression results are largely consistent between sample types, particularly at the pathway level (78, 79). However, for both this reason and the relatively small size of the non–COVID-19 sepsis cohort, these exploratory findings warrant further study.

While severe COVID-19 is less common now than in the beginning of the pandemic, there is still considerable mortality with each wave (80). Simple, rapid prognostic tests for COVID-19 could not only aid in clinical triage and resource allocation during surges but could also identify high-risk patients who may benefit from early targeted interventions (8, 24). Reducing the number of gene targets substantially decreases the technical and computational complexity of host gene expression tests, as well as their cost, making clinical implementation more feasible, especially in resource-limited settings where risk stratification may be disproportionately needed (81). The strong performance of the single gene *OLAH* in both blood and nasal swab samples makes it particularly attractive for translation, as this gene could be readily incorporated into existing nasal swab SARS-CoV-2 diagnostic assays to additionally enable prognostication. Moreover, utility may extend to other respiratory viral infections beyond COVID-19 (40), though this warrants further study. Importantly, regulatory precedent now exists for host gene expression–based diagnostic tests, with several assays having received FDA clearance in the past few years (82, 83). However, recognizing that host gene expression–based tests are still early in clinical adoption, future work should explore whether the classifier genes demonstrate prognostic utility at the protein level, which may enable development of simpler protein-based assays.

Strengths of our study include a large sample size, a multicenter design, incorporation of multiple different sample types, and a rigorous informatics approach for classifier development. However, our study also has limitations. The primary cohort was recruited before COVID-19 vaccines became widely available; however, we found that our classifiers performed equally well in a cohort inclusive of vaccinated patients. Additionally, our cohorts consisted solely of symptomatic hospitalized patients, leaving uncertainty about whether the classifiers would maintain their performance in outpatient settings. Although our study was conducted across multiple centers within the United States, it remains uncertain how these classifiers would perform in international settings, particularly those with differing healthcare infrastructure, resource availability, and demographics. To select genes for our classifier, we utilized LASSO logistic regression; while this approach effectively identified well-performing parsimonious classifiers in our study, it works by eliminating redundant genes, so it is possible that different sets of genes in IMPACC could have comparable prognostic performance. Finally, while the classifier genes may reflect the underlying mechanism of fatal COVID-19 based on prior literature, further validation and functional studies will be necessary to confirm their causal role.

Taken together, we present a comprehensive transcriptomic characterization of fatal COVID-19, illuminating key pathways associated with severe outcomes and developing parsimonious blood- and nasal swab–based classifiers accurately predict mortality COVID-19 early in illness. Moving forward, future efforts will focus on further validating these classifiers across a broader spectrum of disease severity and in international populations, with the goal of refining model parameters and establishing clinically actionable thresholds. Ultimately, successful translation will require adaption to a point-of-care clinical diagnostic platform and real-world assessment of effects on patient management and outcomes in a clinical trial.

## Methods

### Sex as a biological variable.

Our study examined male and female participants. Classifiers were designed to predict 28-day mortality, an outcome that did not differ by sex in our study.

### Study cohorts and design.

This study primarily leveraged the IMPACC observational cohort, which enrolled a total of 1,164 patients hospitalized for COVID-19 from 20 different US hospitals across 15 hospital systems (84, 85). Biological sample collection, processing, and multi-modal immune profiling followed a standard protocol utilized at core laboratories and by every participating academic institution (84, 85).

For our primary analyses, we included all IMPACC participants who met the following inclusion criteria: (a) had at least 1 nasal swab or PBMC sample collected within the first 48 hours of hospital admission for RNA-Seq, and (b) had an admission SARS-CoV-2 viral load measured by either qPCR or RNA-Seq. Samples that failed RNA-Seq quality control standards (described below) were removed, ultimately leaving 894 total patients included. For patients with multiple available samples that met these criteria, only the earliest nasal swab and PBMC sample were retained. Both PBMC transcriptomic data and SARS-CoV-2 viral load measurements were available for 785 IMPACC participants. Nasal transcriptomic data and SARS-CoV-2 viral load measurements were available for 842 IMPACC participants.

Two external cohorts were also studied. We validated our classifiers in the COMET cohort of patients hospitalized for COVID-19 at UCSF and Zuckerberg San Francisco General (ZSFG) hospitals, San Francisco, California, USA, between 2020 and 2023 (*n* = 137) (41). In addition, we compared our findings against critically ill adults with sepsis due to causes other than COVID-19 who were enrolled in the Early Assessment of Renal and Lung Injury (EARLI) cohort between 2013 and 2018 at UCSF and ZSFG hospitals in San Francisco, California, USA (*n* = 122) (86).

### Standardization of SARS-CoV-2 viral load measurements.

Viral load was measured from nasal swab samples either using either SARS-CoV-2 qPCR (CT) or RNA-Seq (reads-per-million [rPM]), which were highly correlated (*P* < 2 × 10^–16^; Supplemental Figure 6). Because some patients did not qPCR performed, we imputed CT values from rPM using a regression model generated on patients who had both samples available. Specifically, we fit a robust regression model using the lmrob function from the R package robustbase(87) on the log-transformed rpM values and CT data using the formula CT ~ ln(rpM+1) (Supplemental Figure 5). If patients had both viral load measurement types available, the CT measurement was used.

### Host gene expression analysis.

RNA-Seq and alignment against the host transcriptome was performed as previously described (84, 85), and the deidentified, quality-controlled raw gene count files and metadata were obtained from the IMPACC study. We retained samples with at least 10,000 genes and retained protein-coding genes that had a minimum of 10 counts in at least 20% of the samples. Differential expression analyses were performed comparing mortality and survival using the R package limma using quantile normalization and the voom method (88, 89), and age and sex were included as covariates. The eBayes function with default parameters was employed to compute empirical Bayes statistics and calculate the *P* values, correcting for multiple testing with Benjamini-Hochberg method. *P*_adj_ < 0.05 were considered significant.

Gene set enrichment analysis (GSEA) was performed using the R package fgsea (90), applying REACTOME pathways with a minimum size of 5 genes and a maximum size of 500 genes (91). All genes from the limma differential expression analyses were included as input, pre-ranked in descending order using the equation –log_10_(*P*_adj_ value) × sign(log_2_[fold change)]. Pathways with *P*_adj_ < 0.05 were considered significant. Identical differential expression and pathway analyses were performed on PBMC and nasal swab RNA-Seq data from IMPACC.

### Comparison of COVID-19 and sepsis mortality.

We compared the biological pathways enriched in COVID-19 mortality to those enriched in sepsis mortality, leveraging whole blood RNA-Seq data from patients with sepsis enrolled in the EARLI cohort (86). Differential expression and pathway analyses comparing survival and mortality in EARLI were performed in the exact same manner as with IMPACC. The top 6 up- and downregulated pathways for COVID-19 and sepsis were selected for visualization. If a pathway did not have GSEA results, the enrichment score was set to zero.

### Development of parsimonious mortality prediction classifiers.

PBMC and nasal swab mortality prediction classifiers were generated separately. For each, patients were first randomly divided into a train set (70%) and test set (30%). Input features for the classifier models included gene expression data, age, and SARS-CoV-2 viral load data. For the train set, we filtered for protein-coding genes with log_2_(fold change) > 1 or < –1 and *P*_adj_ < 0.05 based on the differential expression analysis described previously, in addition to employing standard filtering for protein-coding genes with at least 10 counts in at least 20% of the samples. This gene filter was subsequently applied to the test set. We performed variance-stabilizing transformation on the train set using the R package DESeq2 (92). These dispersion estimates were then applied to the test set. We standardized gene expression, age, and SARS-CoV-2 viral load features in the train set using the caret R package (93) and applied these standardization parameters to the test set.

The train set was used to identify the optimal classifier, and the test set was used to evaluate performance of this classifier. First, the train set was divided into 5 folds for the feature selection step, maintaining a relatively even distribution of mortality across each fold. We methodically iterated through multiple candidate gene sets of varying classifier lengths (ranging from *n* = 2 genes to *n* = 10 genes for PBMC and *n* = 2 genes to *n* = 4 genes for nasal swab, as the latter had far fewer genes that passed filtering). Specifically, for each *n*, we employed the LASSO model in the glmnet R package (94, 95), performing 5-fold cross validation by training the model on 4 of the 5 folds and testing on the remaining fold, ultimately yielding 5 gene lists for each *n* length. Age and SARS-CoV-2 viral load which were included as additional features in each of the classifiers.

We next tested the performance of each candidate classifier (5 classifiers for each gene length *n*) by using repeated random partitioning. Specifically, we performed 50 iterations of randomly splitting the data into 3 folds using the createFolds() function from the caret package (93) and used logistic regression with the classifier features to obtain a distribution of 150 area under the curve (AUC) values for each feature set using the pROC R package (96). The AUC distribution was plotted for each of the best performing classifiers of length *n* (i.e., those with the highest average AUC), as well as for classifiers only incorporating viral load, age, and viral load + age. From these, the classifier with the fewest genes where the average AUC plateaued was chosen as the final classifier.

Finally, we evaluated the performance of these classifiers and a classifier consisting of *OLAH* expression, viral load, and age, on the held-out test set. We fit a logistic regression model on the full train set and made predictions on the test set. Model coefficients for each classifier can be found in (Supplemental Table 6). We generated a ROC curve and calculated AUC and confidence intervals using the pROC package, as described above. We computed a 95% CI for our AUC value using the ci.auc (method = “bootstrap”, boot.n = 5000, boot.stratified = TRUE) function.

### External validation of mortality classifiers.

We externally validated our peripheral blood COVID-19 mortality prediction classifiers in the COMET cohort, which included vaccinated patients. We included all COMET patients that were not co-enrolled in IMPACC and had PBMC available on the day of study enrollment (*n* = 137). Gene expression quality control, filtering, normalization, and transformation were done with identical methods as in IMPACC. Differential expression and GSEA were conducted similarly, controlling for an additional covariate of batch. We evaluated 2 classifiers with different model features: 3 classifier genes and age, and *OLAH* and age. SARS-CoV-2 viral load information was not available in COMET. We retrained 5 logistic regression models using these input features in COMET by employing the same 5-fold cross validation as described previously to generate ROC curves and AUC metrics. We then computed the AUC values on the out-of-fold predictions and bootstrapped as described previously.

### Comparison to clinical prognostic variables and an existing mortality classifier.

As comparators for our classifiers, we evaluated the diagnostic performance of conventional clinical variables (including baseline respiratory ordinal score, SOFA score, CRP, and ALC) and a previously reported 6-gene mortality classifier (*HK3*, *LY86*, *TGFBI*, *DEFA4*, *BATF*, and *HLA-DPB1*) that was originally developed in patients with sepsis (28). For each, we trained logistic regression models in the train set and made predictions on the test set to evaluate performance in IMPACC, implementing the same methodology described above for the 3-gene signature and *OLAH* classifiers.

### Statistics.

Differential expression analyses were performed using the limma package in R, which applies an parametric empirical Bayes moderated *t* statistic (88). Pathway analyses were conducted using the fgsea package, which employs a nonparametric, permutation-based approach to evaluate whether predefined gene sets are enriched (90). Both tests were 2-sided, and *P* values were adjusted for multiple comparisons using the Bejamini-Hochberg method, with an *P*_adj_ < 0.05 considered significant. For evaluation of the host gene expression classifiers, AUC was the main performance metric, and the methodology for classifier development is described in detail in the corresponding Methods section. Statistical significance between ROC curves was determined using DeLong test. The Mann-Whitney *U* test and Fisher’s exact test were used for demographic and clinical variables, as described in the legend for Table 1. All statistical analyses were performed using R (version 4.3.1).

### Study approval.

The Department of Health and Human Services Office for Human Research Protections determined that the IMPACC study protocol met criteria for a public health surveillance exception [45CFR46.102(l) (2)], and the study was approved by each IRB through this exception (12 sites) or by preapproved biobanking protocols (3 sites). The 2 external cohorts, COMET and EARLI, were both approved by the UCSF IRB (protocol nos. 20-30497 and 10-02852, respectively).

### Data availability.

Data used in this study are available at ImmPort Shared Data under the accession no. SDY1760 and in the NLM’s Database of Genotypes and Phenotypes (dbGaP) under the accession no. phs002686.v2.p2. All code is deposited in the following Bitbucket repository: https://bitbucket.org/kleinstein/impacc-public-code/src/master/mortality_prediction_manuscript/ Values for all data points in graphs are reported in the Supporting Data Values file.

## Author contributions

ECL, RN, HVP, and CRL conceived the idea for the project. MCA, SB, WE, NDJ, and SKS generated the data. The IMPACC Network, COMET Consortium, and EARLI Consortium contributed to cohort design, participant enrollment, sample collection, data generation, and/or data quality assurance. RN, ECL, HVP, NS, LPN, and AH analyzed the data. RN, ECL, HVP, NS, LPN, JDA, PMB, SKS, AH, HP, PVZ, CBC, MCA, ADA, SB, WE, LG, NDJ, SHK, FK, HTM, AO, BP, NR, RRM, ER, JS, HS, OL, SCH, DE, CMH, MFK, MAM, PW, EKH, CSC, and CRL provided input on analyses and findings. RN, ECL, HVP, and CRL wrote the manuscript. RN, ECL, HVP, NS, LPN, JDA, PMB, SKS, AH, HP, PVZ, CBC, MCA, ADA, SB, WE, LG, NDJ, SHK, FK, HTM, AO, BP, NR, RRM, ER, JS, HS, OL, SCH, DE, CMH, MFK, MAM, PW, EKH, CSC, and CRL reviewed and edited the manuscript.

## Funding support

This work is the result of NIH funding, in whole or in part, and is subject to the NIH Public Access Policy. Through acceptance of this federal funding, the NIH has been given a right to make the work publicly available in PubMed Central.

- United States National Institutes of Health: (5R01AI135803-03, R35HL140026, 5U19AI118608-04, 5U19AI128910-04, 4U19AI090023-11, 4U19AI118610-06, R01AI145835-01A1S1, 5U19AI062629-17, 5U19AI057229-17, 5U19AI125357-05, 5U19AI128913-03, 3U19AI077439-13, 5U54AI142766-03, 5R01AI104870-07, 3U19AI089992-09, 3U19AI128913-03, 5T32DA018926-18, and K0826161611).
- NIAID, NIH (3U19AI1289130, U19AI128913-04S1, and R01AI122220).
- NCATS (UM1TR004528).
- The National Science Foundation (DMS2310836).
- The Chan Zuckerberg Biohub San Francisco.

## Supplementary Material

Supplemental data

ICMJE disclosure forms

Supplemental data set 1

Supplemental data set 2

Supplemental data set 3

Supplemental data set 4

Supporting data values

## Figures and Tables

**Table 1:**
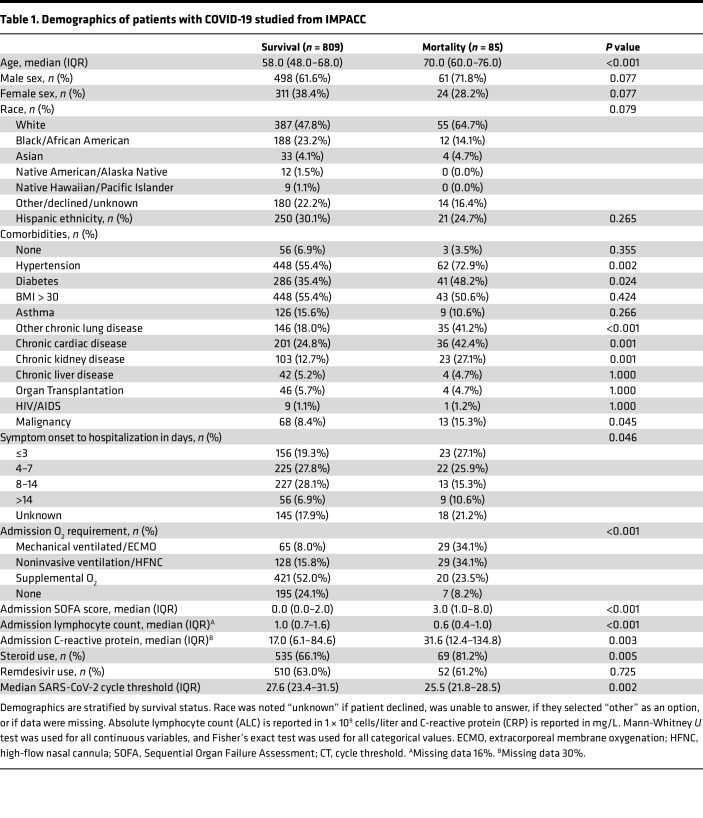
Demographics of patients with COVID-19 studied from IMPACC

## Version 1 11/10/2025

Electronic publication
