# Supplementary material for: Minimalistic transcriptomic signatures permit accurate early prediction of COVID-19 mortality
Source: JCI Insight. 2025 Nov 10;10(21):e195436. doi: 10.1172/jci.insight.195436 (PMC12643502; doi:10.1172/jci.insight.195436)
Supplement: Supplemental data [file jciinsight-10-195436-s147.pdf]

SUPPLEMENTARY MATERIALS

1. **SUPPLEMENTARY FIGURES**
2. **SUPPLEMENTARY TABLES**
3. **SUPPLEMENTARY DATA FILES**
4. **SUPPLEMENTARY ACKNOWLEDGEMENTS**
5. **Conflict of Interest statement**

SUPPLEMENTARY FIGURES

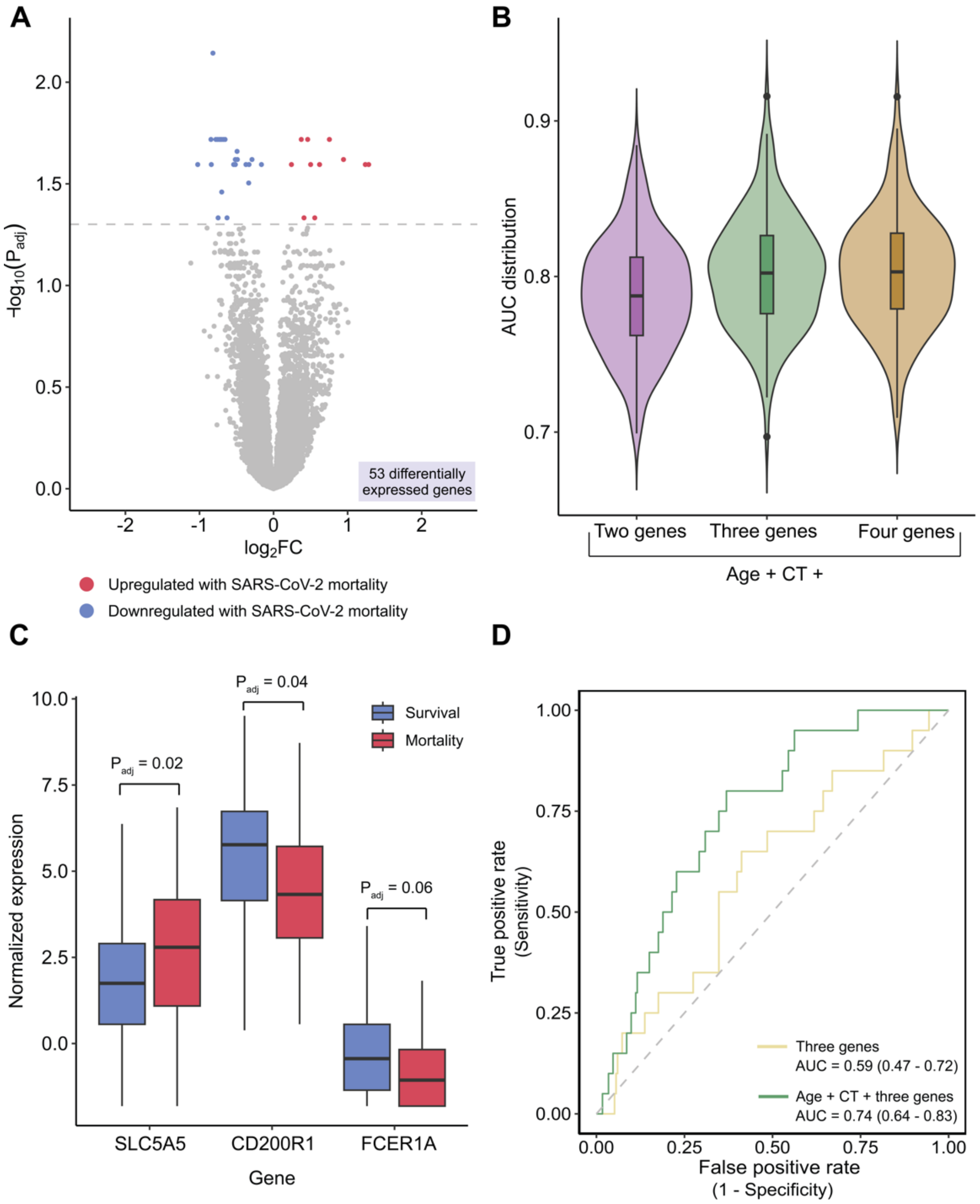

Figure S1. Upper respiratory tract differential expression and host-viral classifier development and evaluation. A. Volcano plot demonstrating the 53 differentially expressed genes between mortality and survival in the upper respiratory tract in the IMPACC cohort (red = upregulated with mortality, blue = downregulated with mortality), using a Benjamini-Hochberg adjusted p-value of 0.05. **B.** Violin plots showing the area under the curve (AUC) distribution for each of the upper respiratory tract classifiers, evaluated in the training cohort. **C.** Boxplots comparing the $\log_2$ counts per million normalized gene expression of the three-gene classifier genes *(SLC5A5, CD200R1*, *FCER1A*) between survival (blue) and mortality (red) in the IMPACC upper respiratory tract data set. **D**. Performance of three-gene classifier with (green) and without (yellow) the addition of age and CT value in the validation cohort. Area under the curve (AUC) listed as value with 95% confidence interval**.**

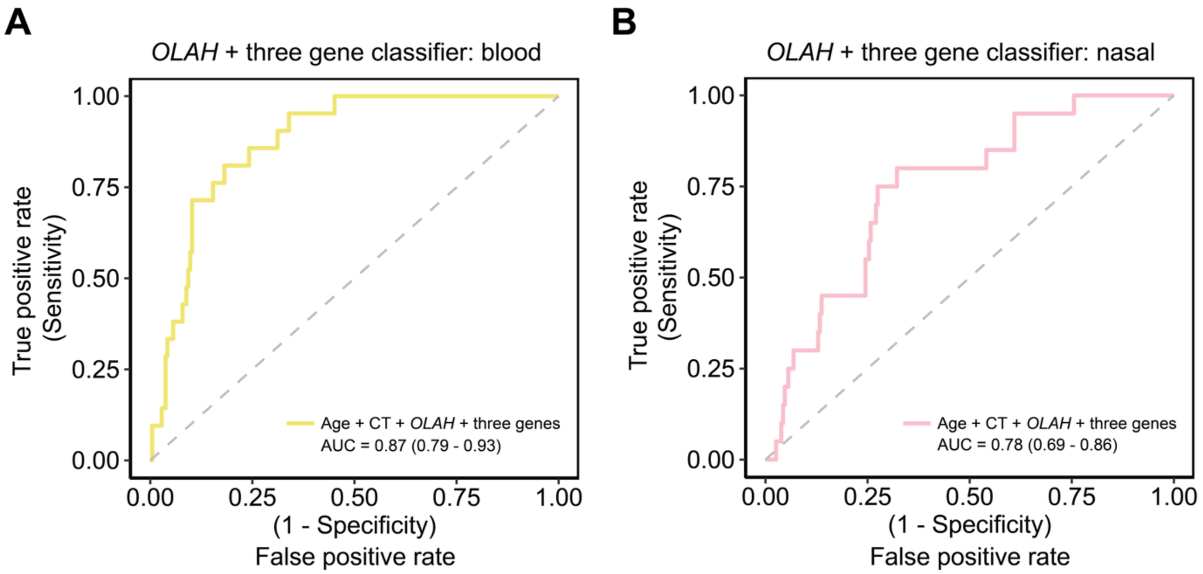

Figure S2. Performance of combined prognostic classifiers in the IMPACC cohort. A. Receiver operating characteristic (ROC) curve displaying the performance of a combined classifier consisting of *OLAH,* the three genes from the blood prognostic classifier, age, and SARS-CoV-2 cycle threshold (CT) in the IMPACC peripheral blood data. Area under the curve (AUC) listed as a value with 95% confidence interval. **B.** ROC curve displaying performance of a classifier consisting of *OLAH,* the three genes from the nasal prognostic classifier, age, and CT.

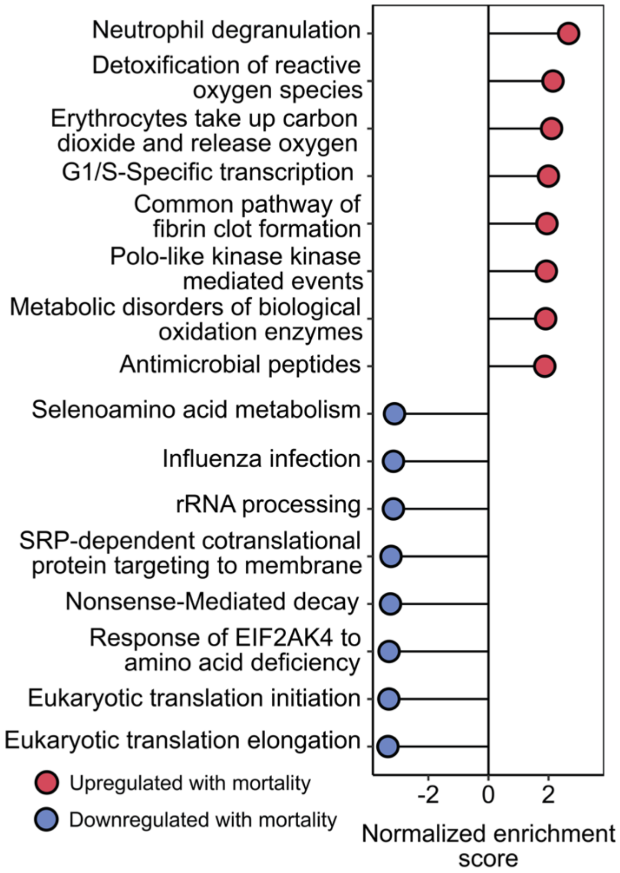

Figure S3. Pathways associated with mortality in the validation cohort. Gene set enrichment analysis (GSEA) demonstrating statistically significant pathways based on Benjamini-Hochberg adjusted p-value in the validation cohort (red = upregulated pathways, blue = downregulated pathways).

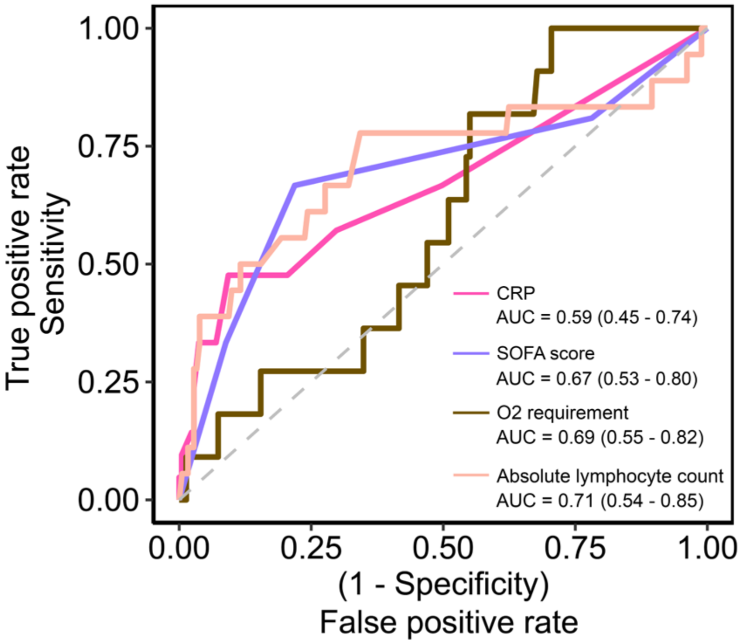

Figure S4. Performance of classifiers consisting of clinical variables in the IMPACC cohort. Receiver operating characteristic (ROC) curve displaying the performance of classifiers consisting of C-reactive protein (CRP), sepsis-related organ failure assessment score (SOFA) score, baseline respiratory ordinal score (O2 requirement), and absolute lymphocyte count. Area under the curve (AUC) listed as a value with 95% confidence intervals. Analysis was performed on individuals with PBMC RNA-seq data available.

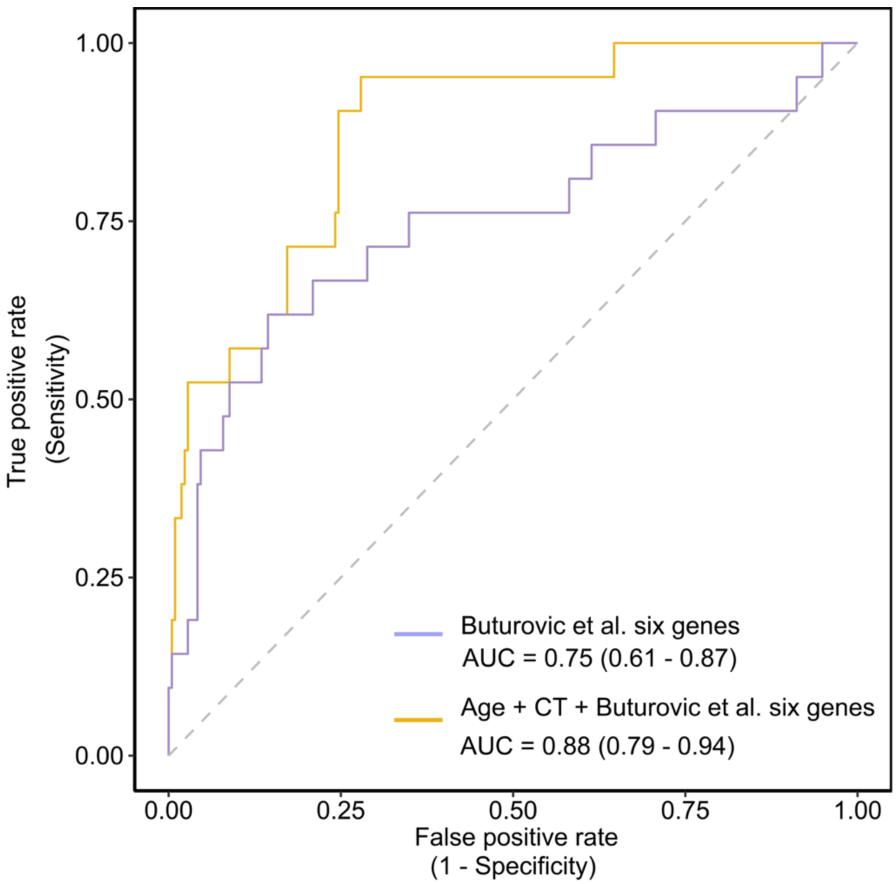

Figure S5. Performance of a previously published mortality classifier in the IMPACC cohort. Performance of the Buturovic et al. six gene classifier with (yellow) and without (purple) the added features of age and SARS-CoV-2 cycle threshold in IMPACC. Area under the curve (AUC) listed as value with 95% confidence interval**.**

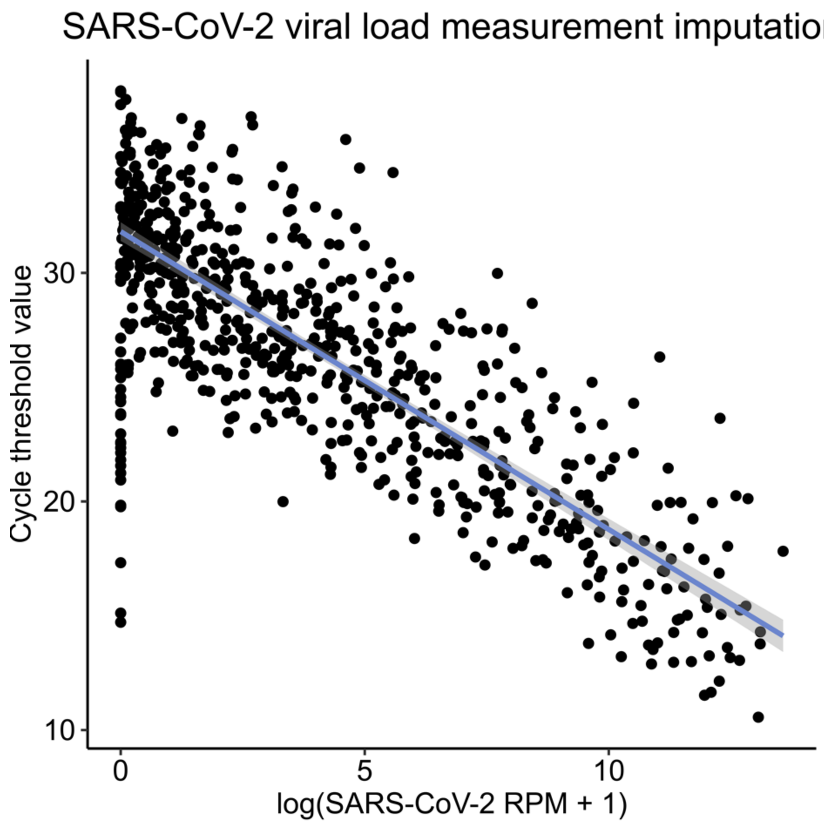

Figure S6. Robust regression for integrating SARS-CoV-2 reads-per-million (RPM) and cycle threshold values. Scatter plot showing the relationship between log(SARS-CoV2 RPM + 1) and SARS-CoV-2 cycle threshold (CT), for all subjects with both data available (n = 708). The line of best fit is based on a robust regression model (adjusted R-squared = 0.693, residual standard error = 2.978). Both the intercept and rpM value were significantly associated with CT value ($P < 2e-16$) based on linear regression.

SUPPLEMENTARY TABLES

	Survival (n=80)	Mortality (n=42)	P-value
**Age, median (IQR)**	65.5 (54.0-74.5)	69.5 (58.0-79.0)	0.234
**Male, n (%)**	48 (60.0%)	28 (66.7%)	0.557
**Female, n (%)**	32 (40.0%)	14 (33.3%)	0.557
**Race, n (%)**			0.722
**White**	34 (42.5%)	13 (31.0%)	
**Black/African American**	11 (13.8%)	7 (16.7%)	
**Asian**	22 (27.5%)	14 (33.3%)	
**American Indian/Alaska Native**	1 (1.3%)	0 (0.0%)	
**Unknown**	12 (15.0%)	8 (19.0%)	
**Hispanic ethnicity, n (%)**	9 (11.3%)	6 (14.3%)	0.762
**Comorbidities, n (%)**			
**Diabetes**	25 (31.3%)	15 (35.7%)	0.686
**Hypertension**	31 (38.8%)	14 (33.3%)	0.693
**Chronic pulmonary disease**	20 (25.0%)	8 (19.0%)	0.505
**Cancer**	13 (16.3%)	10 (23.8%)	0.337
**Immunocompromised**	6 (6.5%)	7 (16.7%)	0.133
**Autoimmune disease**	3 (3.8%)	0 (0.0%)	0.551
**Mechanical ventilation, n (%)**	65 (81.3%)	35 (83.3%)	0.999
**Vasopressor use, n (%)**	62 (77.5%)	37 (88.1%)	0.223

Table S1: Sepsis cohort (EARLI) demographics. Demographics are stratified by survival status. Race was noted “unknown” if patient declined, was unable to answer, if they selected “other” as an option, or if data were missing. Mann-Whitney test was used for all continuous variables, and Fisher’s exact test was used for all categorical values. IQR, interquartile range; EARLI, Early Assessment of Renal and Lung Injury.

Size	Genes	AUC Mean	AUC Std. Dev.
2	***ATP1B2***, ***DAAM2***	0.79	0.04
3	***ATP1B2***, ***DAAM2***, ***CD83***	0.80	0.04
4	***ATP1B2***, ***DAAM2***, *TSPAN7, KCNH8*	0.81	0.04
5	***ATP1B2***, ***DAAM2***, *TSPAN7, KCNH8*, ***CD83***	0.82	0.04
6	***ATP1B2***, ***DAAM2***, *TSPAN7, KCNH8*, ***CD83***, *FCER1A*	0.82	0.04
8	***ATP1B2***, ***DAAM2***, *TSPAN7, KCNH8*, ***CD83***, *FCER1A, NR4A3, CPA3*	0.82	0.05
10	***ATP1B2***, ***DAAM2***, *TSPAN7, KCNH8*, ***CD83***, *FCER1A, NR4A3, CPA3, TMEM45A, MLNR*	0.82	0.04

Table S2. Peripheral blood candidate mortality classifiers generated by LASSO. The best performing gene sets for feature lengths n = 2-6, 8, and 10 based on the 3-fold random partitioning evaluation of performance characteristics. Bolded genes are the genes included in the final three gene classifier. LASSO, least absolute shrinkage and selection operator; AUC, area under the receiver operating characteristic curve.

Length	Genes	AUC mean	AUC Std. Dev
2	***SLC5A5, CD200R1***	0.79	0.04
3	***SLC5A5, CD200R1**, **FCER1A***	0.80	0.04
4	***SLC5A5, CD200R1**, **FCER1A**, MARCO*	0.80	0.04

Table S3. Nasal swab candidate mortality classifiers generated by LASSO. The best performing gene sets for feature lengths n = 2-4 based on the 3-fold random partitioning evaluation of performance characteristics. Bolded genes are the genes included in the final three gene classifier. LASSO, least absolute shrinkage and selection operator; AUC, area under the receiver operating characteristic curve.

	Survival (n=116)	Mortality (n=21)	P-value
**Age, median (IQR)**	56.0 (44.3-70.2)	67.5 (49.9-78.2)	0.052
**Male, n (%)**	78 (67.2%)	13 (61.9%)	0.625
**Female, n (%)**	38 (32.8%)	8 (38.1%)	0.625
**Race, n (%)**			0.391
**White**	38 (32.8%)	6 (28.6%)	
**Black/African American**	10 (8.6%)	5 (23.8%)	
**Asian**	18 (15.5%)	4 (19.0%)	
**Native Hawaiian/Pacific Islander**	2 (1.7%)	0 (0.0%)	
**American Indian/Alaska Native**	1 (0.9%)	0 (0.0%)	
**Unknown**	47 (40.5%)	6 (28.6%)	
**Hispanic ethnicity, n (%)**	48 (41.4%)	8 (38.1%)	0.815
**Comorbidities, n (%)**			
**BMI > 30**	51 (44.0%)	10 (47.6%)	0.814
**HIV**	3 (2.6%)	1 (4.8%)	0.490
**Rheumatologic disorder**	15 (12.9%)	2 (9.5%)	0.999
**Solid organ transplant**	17 (14.7%)	2 (9.5%)	0.737
**Mechanical ventilation, n (%)**	34 (29.3%)	16 (76.2%)	<0.001
**Steroid use, n (%)**	59 (50.9%)	17 (81.0%)	0.016
**Vaccinated for COVID-19, n (%)**	45 (38.8%)	10 (47.6%)	0.476

Table S4: COVID-19 external validation cohort (COMET) demographics. Demographics are stratified by survival status. Race was noted "unknown" if patient declined, was unable to answer, if they selected "other" as an option, or if data were missing. Mann-Whitney test was used for all continuous variables, and Fisher's exact test was used for all categorical values. IQR, interquartile range; BMI, body mass index; COMET, COVID-19 Multi-immunophenotyping projects for Effective Therapies.

SOFA classifier	AUC (95% CI)	Comparison classifier	AUC (95% CI)	DeLong P-value
SOFA	0.67 (0.51 - 0.80)	Three genes + Age + CT (PBMC)	0.88 (0.82 - 0.94)	0.003
SOFA	0.67 (0.51 - 0.80)	OLAH + Age + CT (PBMC)	0.86 (0.79 - 0.93)	0.010
SOFA	0.73 (0.61 - 0.83)	Three genes + Age + CT (NS)	0.74(0.64 - 0.83)	0.400
SOFA	0.73 (0.61 - 0.83)	OLAH + Age + CT (NS)	0.78 (0.69 - 0.86)	0.064

Table S5: Comparison between performance of a prognostic model based on Sepsis-related Organ Failure Assessment (SOFA) score and parsimonious classifiers. Performance is based on analysis in the respective cohort (PBMC or NS). Significance between the area under the curve (AUC) values was determined based on the paired DeLong test. PBMC, peripheral blood mononuclear cell; NS, nasal swab.

Model	Feature	Coefficients
PBMC 3 gene + age + CT	*CD83*	-0.52
	ATP1B2	0.54
	DAAM2	0.45
	Age	0.66
	CT	-0.28
PBMC *OLAH* + age + CT	*OLAH*	0.42
	Age	0.68
	CT	-0.30
NS 3 gene + age + CT	*SLC5A5*	0.57
	CD200R1	-0.51
	FCER1A	0.47
	Age	0.75
	CT	-0.25
NS *OLAH* + age + CT	*OLAH*	0.44
	Age	0.82
	CT	-0.36

Table S6. Model coefficients for features for the four main models discussed in the main manuscript. Each model consisted of a combination of genes, age, and SARS-CoV-2 cycle threshold value. Coefficients are generalized linear model coefficients with a logistic regression binary task. PBMC, peripheral blood mononuclear cell; CT, cycle threshold.

SUPPLEMENTARY DATA FILES

Supplementary Data File 1. **A.** Genes differentially expressed with mortality in IMPACC blood samples (n=785), adjusted for age and sex. **B.** Gene set enrichment analysis (GSEA) of differentially expressed genes. Legend: logFC = log(2) fold change; padj = Benjamini-Hochberg adjusted P value; NES = normalized enrichment score; size = size of Reactome pathway; LeadingEdge = leading edge genes.

Supplementary Data File 2. **A.** Genes differentially expressed with mortality in IMPACC upper respiratory tract samples (n=842), adjusted for age and sex . **B.** Gene set enrichment analysis (GSEA) of differentially expressed genes. Legend: logFC = log(2) fold change; padj = Benjamini-Hochberg adjusted P value; NES = normalized enrichment score; size = size of Reactome pathway; LeadingEdge = leading edge genes.

Supplementary Data File 3. **A.** Genes differentially expressed with mortality in EARLI blood samples (n=122), adjusted for age and sex. **B.** Gene set enrichment analysis (GSEA) of differentially expressed genes. Legend: logFC = log(2) fold change; padj = Benjamini-Hochberg adjusted P value; NES = normalized enrichment score; size = size of Reactome pathway.

Supplementary Data File 4. **A.** Genes differentially expressed with mortality in COMET blood samples (n=137), adjusted for age and sex. **B.** Gene set enrichment analysis (GSEA) of differentially expressed genes. Legend: logFC = log(2) fold change; padj = Benjamini-Hochberg adjusted P value; NES = normalized enrichment score; size = size of Reactome pathway; LeadingEdge = leading edge genes.

SUPPLEMENTARY ACKNOWLEDGEMENTS

A. IMPACC Network Consortium

Elaine F. Reed, Joanna Schaenman, Ramin Salehi-rad, Adreanne M. Rivera, Harry Pickering, Subha Sen, David Elashoff, Dawn C. Ward, Jenny Brook, Estefania Ramires-Sanchez, Megan Llamas, Claudia Perdomo, Clara E. Magyar, Jennifer Fulcher, David J. Erle, Carolyn S. Calfee, Carolyn M. Hendrickson, Kirsten N. Kangelaris, Viet Nguyen, Deanna Lee, Suzanna Chak, Rajani Ghale, Ana Gonzalez, Alejandra Jauregui, Carolyn Leroux, Luz Torres Altamirano, Ahmad Sadeed Rashid, Andrew Willmore, Prescott G. Woodruff, Matthew F. Krummel, Sidney Carrillo, Alyssa Ward, Charles R. Langelier, Ravi Patel, Michael Wilson, Ravi Dandekar, Bonny Alvarenga, Jayant Rajan, Walter Eckalbar, Andrew W. Schroeder, Gabriela K. Fragiadakis, Alexandra Tsitsiklis, Eran Mick, Yanedth Sanchez Guerrero, Christina Love, Lenka Maliskova, Michael Adkisson, Aleksandra Leligdowicz, Alexander Beagle, Arjun Rao, Austin Sigman, Bushra Samad, Cindy Curiel, Cole Shaw, Gayelan Tietje-Ulrich, Jeff Milush, Jonathan Singer, Joshua J. Vasquez, Kevin Tang, Legna Betancourt, Lekshmi Santhosh, Logan Pierce, Maria Tecero Paz, Michael M. Matthay, Neeta Thakur, Nicklaus Rodriguez, Nicole Sutter, Norman Jones, Pratik Sinha, Priya Prasad, Raphael Lota, Sadeed Rashid, Saurabh Asthana, Sharvari Bhide, Tasha Lea, Yumiko Abe-Jones, Lauren I. R. Ehrlich, Esther Melamed, Cole Maguire, Dennis Wylie, Justin F. Rousseau, Kerin C. Hurley, Janelle N. Geltman, Nadia Siles, Jacob E. Rogers, Pablo Guaman Tipan, Nadine Rouphael, Steven E. Bosinger, Arun K. Boddapati, Greg K. Tharp, Kathryn L. Pellegrini, Brandi Johnson, Bernadine Panganiban, Christopher Huerta, Evan J. Anderson, Hady Samaha, Jonathan E. Sevransky, Laurel Bristow, Elizabeth Beagle, David Cowan, Sydney Hamilton, Thomas Hodder, Amer Bechnak, Andrew Cheng, Aneesh Mehta, Caroline R. Ciric, Christine Spainhour, Erin Carter, Erin M. Scherer, Jacob Usher, Kieffer Hellmeister, Laila Hussaini, Lauren Hewitt, Nina Mcnair, Susan Pereira Ribeiro, Sonia Wimalasena, Jordan P. Metcalf, Nelson I. Agudelo Higuita, Lauren A. Sinko, J. Leland Booth, Douglas A. Drevets, Brent R. Brown, Mark A. Atkinson, Scott C. Brakenridge, Ricardo F. Ungaro, Brittany Roth Manning, Lyle Moldawer, William B. Messer, Catherine L. Hough, Sarah A. R. Siegel, Peter E. Sullivan, Zhengchun Lu, Amanda E. Brunton, Matthew Strand, Zoe L. Lyski, Felicity J. Coulter, Courtney Micheletti, Matthew C. Altman, Naresh Doni Jayavelu, Scott Presnell, Bernard Kohr, Tomasz Jancsyk, Azlann Arnett, Patrice M. Becker, Alison D. Augustine, Steven M. Holland, Lindsey B. Rosen, Serena Lee, Tatyana Vaysman, Al Ozonoff, Joann Diray-Arce, Jing Chen, Alvin T. Kho, Carly E. Milliren, Annmarie Hoch, Ana C. Chang, Kerry McEnaney, Caitlin Syphurs, Brenda Barton, Claudia Lentucci, Maimouna D. Murphy, Mehmet Saluvan, Tanzia Shaheen, Shanshan Liu, Marisa Albert, Arash Nemati Hayati, Robert Bryant, James Abraham, Mitchell Cooney, Meagan Karoly, Ofer Levy, Hanno Steen, Patrick van Zalm, Benoit Fatou, Kinga K. Smolen, Arthur Viode, Simon van Haren, Meenakshi Jha, David Stevenson, Sanya Thomas, Boryana Petrova, Naama Kanarek, Ana Fernandez-Sesma, Viviana Simon, Florian Krammer, Harm Van Bakel, Seunghee Kim-Schulze, Ana Silvia Gonzalez-Reiche, Jingjing Qi, Brian Lee, Juan Manuel Carreño, Gagandeep Singh, Ariel Raskin, Johnstone Tcheou, Zain Khalil, Adriana van de Guchte, Keith Farrugia, Zenab Khan, Geoffrey Kelly, Komal Srivastava, Lily Q. Eaker, Maria C. Bermúdez-González, Lubbertus C. F. Mulder, Katherine F. Beach, Miti Saksena, Deena Altman, Erna Kojic, Levy A. Sominsky,

Arman Azad, Dominika Bielak, Hisaaki Kawabata, Temima Yellin, Miriam Fried, Leeba Sullivan, Sara Morris, Giulio Kleiner, Daniel Stadlbauer, Jayeeta Dutta, Hui Xie, Manishkumar Patel, Kai Nie, Brian Monahan, David A. Hafler, Ruth R. Montgomery, Albert C. Shaw, Steven H. Kleinstein, Jeremy P. Gygi, Dylan Duchen, Shrikant Pawar, Anna Konstorum, Ernie Chen, Chris Cotsapas, Xiaomei Wang, Charles Dela Cruz, Akiko Iwasaki, Subhasis Mohanty, Allison Nelson, Yujiao Zhao, Shelli Farhadian, Hiromitsu Asashima, Omkar Chaudhary, Andreas Coppi, John Fournier, M. Catherine Muenker, Khadir Raddassi, Michael Rainone, William Ruff, Syim Salahuddin, Wade L. Shulz, Pavithra Vijayakumar, Haowei Wang, Esio Wunder Jr., H. Patrick Young, Albert I. Ko, Gisela Gabernet, Denise Esserman, Leying Guan, Anderson Brito, Jessica Rothman, Nathan D. Grubaugh, Kexin Wang, Leqi Xu, Holden Maecker, Bali Pulendran, Kari C. Nadeau, Yael Rosenberg-Hasson, Michael Leipold, Natalia Sigal, Angela Rogers, Andrea Fernandes, Monali Manohar, Evan Do, Iris Chang, Alexandra S. Lee, Catherine Blish, Henna Naz Din, Jonasel Roque, Linda N. Geng, Maja Artandi, Mark M. Davis, Neera Ahuja, Samuel S. Yang, Sharon Chinthrajah, Thomas Hagan, Tyson H. Holmes, Koji Abe, Lindsey R. Baden, Kevin Mendez, Jessica Lasky-Su, Alexandra Tong, Rebecca Rooks, Michael Desjardins, Amy C. Sherman, Stephen R. Walsh, Xhoi Mitre, Jessica Cauley, Xiaofang Li, Bethany Evans, Christina Montesano, Jose Humberto Licona, Jonathan Krauss, Nicholas C. Issa, Jun Bai Park Chang, Natalie Izaguirre, David B. Corry, Farrah Kheradmand, Li-Zhen Song, Ebony Nelson, Monica Kraft, Chris Bime, Jarrod Mosier, Heidi Erickson, Ron Schunk, Hiroki Kimura, Michelle Conway, Dave Francisco, Allyson Molzahn, Connie Cathleen Wilson, Ron Schunk, Trina Hughes, Bianca Sierra, Jordan Oberhaus, Faheem W. Guirgis, Brittney Borresen, Matthew L. Anderson, Bjoern Peters, James A. Overton, Randi Vita, Kerstin Westendorf, Scott J. Tebbutt, Casey P. Shannon, Rafick-Pierre Sekaly, Slim Fourati, Grace A. McComsey, Paul Harris, Scott Sieg, George Yendewa, Mary Consolo, Heather Tribout, Susan Pereira Ribeiro, Charles B. Cairns, Elias K. Haddad, Michele A. Kutzler, Mariana Bernui, Gina Cusimano, Jennifer Connors, Kyra Woloszczuk, David Joyner, Carolyn Edwards, Edward Lee, Edward Lin, Nataliya Melnyk, Debra L. Powell, James N. Kim, I. Michael Goonewardene, Brent Simmons, Cecilia M. Smith, Mark Martens, Brett Croen, Nicholas C. Semenza, Mathew R. Bell, Sara Furukawa, Renee McLin, George P. Tegos, Brandon Rogowski, Nathan Mege, Kristen Ulring, Pam Schearer, Judie Sheidy, Crystal Nagle, James A. Overton, Scott R. Hutton, Greg Michelotti, Kari Wong, Adeeb Rahman & Vicki Seyfert-Margolis.

B. COMET Consortium

K. Mark Ansel, Stephanie Christenson, Michael Adkisson, Walter Eckalbar, Lenka Maliskova, Andrew Schroeder, Raymund Bueno, Gracie Gordon, George Hartoularos, Divya Kushnoor, David Lee, Elizabeth McCarthy, Anton Ogorodnikov, Matthew Spitzer, Kamir Hiam, Yun S. Song, Yang Sun, Erden Tumurbaatar, Monique van der Wijst, Alexander Whatley, Chayse Jones, Saharai Caldera, Catherine DeVoe, Paula Hayakawa Serpa, Christina Love, Eran Mick, Maira Phelps, Alexandra Tsitsiklis, Carolyn Leroux, Sadeed Rashid, Nicklaus Rodriguez, Kevin Tang, Luz Torres Altamirano, Aleksandra Leligdowicz, Michael Matthay, Michael Wilson, Jimmie Ye, Suzanna Chak, Rajani Ghale, Alejandra Jauregui, Deanna Lee, Viet Nguyen, Austin Sigman, Kirsten N. Kangelaris, Saurabh Asthana, Zachary Collins, Ravi Patel, Arjun Rao, Bushra Samad, Cole Shaw, Andrew Willmore, Tasha Lea, Gabriela K. Fragiadakis, Carolyn S. Calfee, David J.

Erle, Carolyn M. Hendrickson, Matthew F. Krummel, Charles R. Langelier, Prescott G. Woodruff, Sidney C. Haller, Alyssa Ward, Norman Jones, Jeff Milush, Vincent Chan, Nayvin Chew, Alexis Combes, Tristan Courau, Kenneth Hu, Billy Huang, Nitasha Kumar, Salman Mahboob, Priscila Muñoz-Sandoval, Randy Parada, Gabriella Reeder, Alan Shen, Jessica Tsui, Shoshana Zha & Wandi S. Zhu.

C. EARLI Consortium

Narges Alipanah-Lechner, Kim Bardillon, Carolyn S. Calfee, Suzanna Chak, Olivia Chao Sidney A. Carrillo, Taarini Hariharan, Carolyn M. Hendrickson, Charles R Langelier, Deanna Lee, Carolyn Leroux, Chelsea Lin, Michael A. Matthay, Lucile P. A. Neyton, Angelika Ringor, Aartik Sarma, Emma Schmiege, Natasha Spottiswoode, Kathryn M. Sullivan, Melanie F Weingart, Andrew Willmore, Hanjing Zhuo.

Conflict of interest: The Icahn School of Medicine at Mount Sinai has filed patent applications relating to SARS-CoV-2 serological assays, NDV-based SARS-CoV-2 vaccines influenza virus vaccines, and influenza virus therapeutics which list FK as coinventor and he has received royalty payments from some of these patents, US Patents 20250228929A1, 20250186578A1, and 20250041402A1. Mount Sinai has spun out a company, Kantaro, to market serological tests for SARS-CoV-2 and another company, Castlevax, to develop SARS-CoV-2 vaccines, and FK is cofounder and scientific advisory board member. FK has consulted for Merck, GSK, Sanofi, Curevac, Seqirus, and Pfizer and is currently consulting for 3rd Rock Ventures, Gritstone and Avimex, as well as collaborating with Dynavax on influenza vaccine development and with VIR on influenza virus therapeutics. OL is a named inventor on US patents 12268739 and 11730810 held by Boston Children's Hospital relating to vaccine adjuvants and human in vitro platforms that model vaccine action. His laboratory has received research support from GlaxoSmithKline (GSK), where he is a consultant. He is also a cofounder of and advisor to ARMR Sciences. CBC serves as a consultant to bioMerieux and is funded for a grant from Bill & Melinda Gates Foundation. JAO is a consultant at Knocean Inc. JLS serves as a scientific advisor of Precion Inc. SRH, GM, and KW are employees of Metabolon Inc. VSM is a current employee of MyOwnMed. NR reports grants or contracts with Merck, Sanofi, Pfizer, Vaccine Company, Quidel, Lilly, and Immorna and has participated on data safety monitoring boards for Moderna, Sanofi, Seqirus, Pfizer, EMMES, ICON, BARDA, Imunon, CyanVac, and Micron. NR has also received support for meetings/travel from Sanofi and Moderna and honoraria from Virology Education.
